# Patients' Experience of Anxiety and Pain during Retrobulbar Injections prior to Vitrectomy

**DOI:** 10.1155/2019/8098765

**Published:** 2019-07-31

**Authors:** Michael Mimouni, Hamza Abualhasan, Kamal Mtanes, Fares Mazzawi, Yoreh Barak

**Affiliations:** ^1^Department of Ophthalmology, Rambam Health Care Campus, Haifa, Israel; ^2^Technion-Israel Institute of Technology, Haifa, Israel

## Abstract

**Purpose:**

The purpose of this study was to evaluate the correlation between pain associated with retrobulbar block and anxiety levels before the injection.

**Methods:**

This prospective observational, noninterventional study included consecutive patients who received a retrobulbar block by a single surgeon prior to undergoing 25G PPV at the Department of Ophthalmology, Rambam Health Care Campus, between April 2016 and August 2017. Patients plotted their anxiety levels (scale 0–10) using the visual analogue scale for anxiety (VASA), and immediately after receiving the injection, they plotted their experienced level of pain (scale 0–10) using the visual analogue scale for pain (VAS), with scores ≥7 defined as severe.

**Results:**

Overall, 48 eyes of 48 patients aged 68.4 ± 10.3 years were included, of which 62.5% were of male gender. Severe anxiety and pain were experienced by 10.4% and 12.5%, respectively. There was a significant correlation between VASA and VAS scores (*r* = 0.43, *p*=0.002) with no other preprocedural parameters demonstrating a significant association with the VAS score. In multivariate analysis, the VASA score was the only factor that was significant (*p*=0.01), and a patient with a severe VASA score was 20 times more likely of experiencing severe pain (*p*=0.006). The ROC curve analysis revealed an area under the curve of 0.89 (*p* < 0.001), and a VASA score >4 demonstrated a sensitivity of 83.3% and a specificity of 73.8% in predicting severe pain.

**Conclusions:**

Approximately 10% of patients experience severe anxiety and pain during retrobulbar blocks. Considering the importance of compliance, reducing anxiety and premedication may be considered, particularly in high-risk patients (VASA score > 4).

## 1. Introduction

Pars plana vitrectomy (PPV) is considered the definitive treatment of choice for vitreous hemorrhage [1], rhegmatogenous retinal detachment (RRD) [2], macular hole [3], epiretinal membrane (ERM) [4], and other indications. Currently, PPV is one of the most common ophthalmic surgical procedures across the world, with over 500,000 vitrectomy surgeries being performed each year [5]. Specifically, small-gauge vitrectomy has gained popularity because of decreased surgical times, less tissue manipulation, and reduced inflammation and pain postoperatively with more rapid visual recovery [6]. Though general anesthesia is the gold standard method of anesthesia for PPV, it is time-consuming and expensive and bears added intra- and postoperative risks. Given the potential advantages of day-care vitrectomy surgery, local anesthetic modalities are of paramount importance. Indeed, it has been established that retrobulbar block is an efficient and safe alternative to general anesthesia in patients undergoing small-gauge PPV [7].

Retrobulbar block carries its own potential complications including dreaded ones, both local (ocular perforation) [8] and systemic (brainstem infarction) [9]. As such, patient cooperation during the injection is critical to ensure safety as sudden movement or the patient looking in the wrong direction could lead to unwanted outcomes [10]. Patient cooperation during ophthalmic procedures may be influenced by several parameters, especially pain experienced throughout the procedure [11]. As such, identifying factors associated with pain and reduction of pain is of interest.

The main purpose of this study was to evaluate the correlation between pain associated with retrobulbar block and anxiety levels before the injection. In addition, we evaluated the relationship between demographic characteristics such as age, gender, and prior injections with preprocedural anxiety and procedure-related pain. Finally, we attempted to characterize patients at high risk for severe pain during retrobulbar block.

## 2. Materials and Methods

The study was carried out with approval from the Rambam Health Care Campus Ethics Board. The study adhered to the tenets set forth in the Declaration of Helsinki. Signed informed consents were collected from all of the recruited patients.

### 2.1. Subjects

All patients included in this prospective consecutive observational, noninterventional study received a retrobulbar block prior to undergoing 25G PPV at the Department of Ophthalmology, Rambam Health Care Campus, between April 1, 2016, and August 31, 2017. All patients were 18 years or older and were referred to PPV by retina specialists in our institution. Indications for injection included vitreous hemorrhage, rhegmatogenous retinal detachment, macular hole, and epiretinal membrane. Exclusion criteria were anterior segment conditions that could affect pain sensation, such as conjunctival irritation, active conjunctivitis, or keratitis or bullous keratopathy or a history of herpetic ocular infection.

### 2.2. Data Collection

Variables recorded were the patient's age, gender, whether or not the patient was a native of the country, level of education (middle school, high school, or university), indication for PPV, whether or not the patient was retired, whether or not the patient had undergone previous PPV, whether or not the patient had undergone previous ocular surgery, and history of use of psychiatric medications (antidepressants, anxiolytics, or antipsychotics). Any use of preprocedural anxiolytic was recorded.

Patients were asked during the routine presurgical intake to plot their anxiety levels using the visual analogue scale for anxiety (VASA) on a scale from 0 to 10 with “no anxiety or fear” scored as 0 and “unbearable anxiety or fear” scored as 10. The visual analogue scales are well-documented and validated tools for reliable assessment of anxiety [12], pain [13], and other variables [14]. The visual analogue scale for pain (VAS) has been used to assess pain in studies in several ophthalmic fields such as refractive surgery [15], cataract surgery [16], vitrectomy [17], and intravitreal injections [18]. In order to ensure correct assessment of anxiety and pain, the patients received an explanation of both VASA and VAS prior to the retrobulbar injection. Immediately after receiving the injection, the subject was asked to plot their experienced level of pain during the injection using a VAS. Anxiety (VASA) and pain (VAS) were further categorized as mild (≤3), moderate (4–6), or severe (≥7).

### 2.3. Surgical Technique

All patients underwent the following retrobulbar injection in a single eye by an experienced surgeon (Y. B.) in the surgery room: after topical instillation of oxybuprocaine hydrochloride 0.4%, skin sterilization was performed with an alcohol pad, and afterwards, a 5 ml mixture of 1 : 1 bupivacaine 0.5% and lidocaine 2% was injected with an Atkinson 23G retrobulbar needle through the inferior lid and into the retrobulbar space.

### 2.4. Statistical Analysis

All data collected in this study were recorded using Microsoft Excel 2013 (Microsoft Corporation). Statistical analyses were performed using Minitab software, version 17 (Minitab Inc, State College, PA). Results were expressed as mean ± SD, median (range), or *N* (%). We compared preinjection characteristics of patients, by using Student's *t*-test for normally distributed variables or Kruskal–Wallis test for nonparametric variables. We used chi-Square or Fisher's exact test as indicated for analysis of categorical variables. One-way analysis of variance (ANOVA) was used for comparison of multiple group averages. Spearman or Pearson's correlation was used to analyze the relationship between preoperative variables and VAS scores wherever appropriate. Stepwise backward multivariate logistic regression analysis was done to determine the baseline variables that enabled to predict VAS scores. For this purpose, we introduced as independent variables those variables that reached a significant level less than 0.30 in univariate analysis. Receiver-operating characteristic (ROC) curve analysis was then performed to identify the optimal VASA score (Youden's index) predicting severe pain (VAS score ≥ 7). A *p* value less than 0.05 was considered statistically significant.

## 3. Results

**Figure 1:**
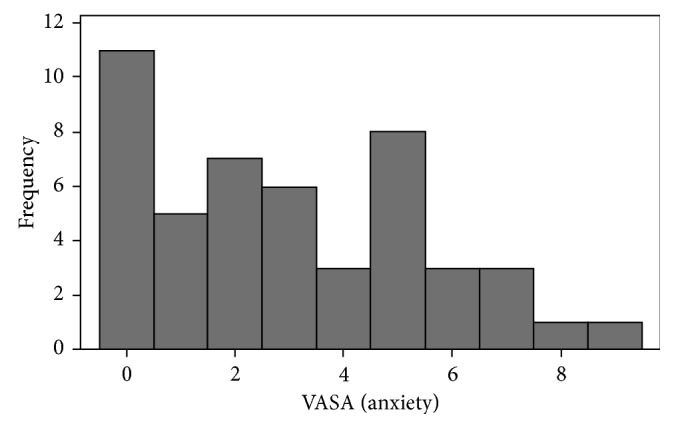
Frequency of different visual analog scale for anxiety (VASA) scores prior to retrobulbar injection for pars plana vitrectomy.

Overall, 48 eyes of 48 patients aged 68.4 ± 10.3 years (range 48–88 years) were included in this study, of which 62.5% were of male gender. The VASA scores were 3.0 ± 2.5 (range 0–9.0) with the majority of patients experiencing mild (60.4%) to moderate (29.1%) anxiety and the rest (10.4%) experiencing severe anxiety (Figure 1).

Table 1 depicts the sensations experienced by the patients during the procedure. The most common sensation was a feeling of pressure (52.1%) followed by a slight pinching pain (27.1%). Most patients described the pain as being more or less than what they expected (62.5%) with 20.8% feeling more pain than expected and 16.7% feeling less. Most of the VAS scores were in the mild range (56.3%) followed by the moderate range (31.3%) and severe range (12.5%).

There were a statistically significant correlation between preprocedural VASA and VAS scores (*r* = 0.43, *p*=0.002) and no significant correlation between age and VAS score (*r* = −0.02, *p*=0.90). The results of the univariate correlational analyses between VAS scores and preprocedural parameters are depicted in Table 2. In brief, other than native (4.4 ± 2.9) versus nonnative (3.0 ± 2.2), where a trend towards significant differences was noted (*p*=0.10), no other parameters were significantly associated with VAS scores.

**Figure 2:**
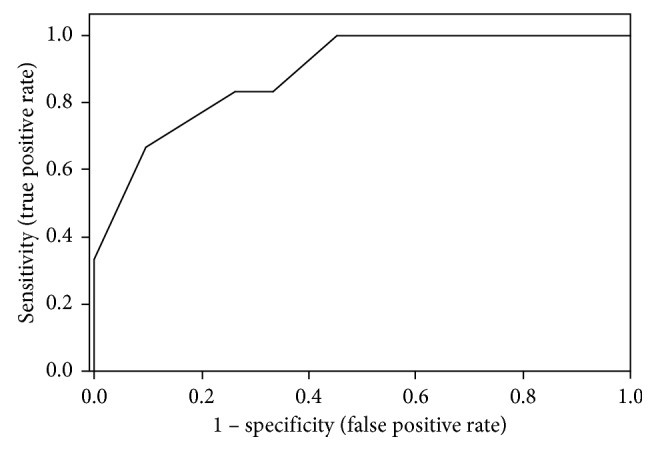
Receiver-operating characteristic curve of visual analog scale for anxiety (VASA) scores as a predictor of severe pain (area under the curve 0.89, *p* < 0.001).

Table 3 depicts the results of the multivariate analysis, in which the VAS score was the dependent variable and all variables that reached *p* < 0.30 in univariate analysis served as independent variables. In brief, the VASA score was the only factor that was significant in multivariate analysis explaining 18.9% of the variance in VAS scores (*p*=0.01). The association between ranges of the VASA score and the odds of a severe VAS score is further detailed in Table 4. In brief, a patient with a severe VASA score was 20 times more likely of experiencing severe pain (*p*=0.006), while a patient with a mild VASA score was 10 times less likely of experiencing severe pain (*p*=0.02). The ROC curve analysis is presented in Figure 2 (AUC = 0.89, *p* < 0.001). In brief, a VASA score >4 demonstrated a sensitivity of 83.3% and a specificity of 73.8% in predicting severe pain (Table 5).

## 4. Discussion

This study assessed the correlation between preoperative parameters and pain from retrobulbar injections. Approximately 10% of patients experienced severe anxiety and pain, and the only parameter significantly associated with pain in univariate analysis was preoperative anxiety, with patients feeling severe anxiety being very likely of experiencing severe pain. In addition, a VASA score >4 was identified as a predictor of severe pain.

Previous studies have evaluated anxiety in patients undergoing ophthalmic procedures. Segal et al. showed that approximately 25% of patients reported high levels of anxiety prior to injections [18]. Similarly, Senra et al. reported that a similar proportion of patients receiving intravitreal injections showed clinical levels of anxiety regardless of the number of injections [19]. Similarly, in the current study, approximately 10% of patients experienced severe anxiety and pain, and another one-third reported moderate levels of anxiety and pain. As such, identifying methods of reducing anxiety is of interest, and indeed, several attempts have been made to reduce anxiety associated with ophthalmic procedures. Chaudhary et al. reported that electronic educational information about intravitreal injections in the waiting room was ineffective at reducing anxiety [20]. Chen et al. showed that classical music before and during intravitreal injections decreased anxiety, though it did not decrease pain [21]. Khezri et al. demonstrated that melatonin and gabapentin reduce anxiety before cataract surgery with retrobulbar block and that gabapentin decreased pain during retrobulbar placement [22]. Rifkin and Schaal reported that gender, older age, and improved vision from previous injection influenced pain experienced during intravitreal injections [23]. Additional interventions reported to have potential to reduce anxiety during ophthalmic procedures include positive suggestions and anxiety management techniques [24], handholding during surgery [25, 26], preoperative midazolam [27], and electronic patient-controlled alert devices.

In the current study, there was a statistically significant correlation between preprocedural VASA and VAS scores (*r* = 0.43, *p*=0.002), with no other parameters significantly associated with the VAS score including multivariate analysis. In addition, patients with a severe VASA score were 20 times more likely to experience severe pain. This finding is supported by Segal et al. who reported a significant correlation between anxiety prior to intravitreal injections and pain during intravitreal injections [18]. Interestingly, Jiang et al. reported that when compared to first eye cataract surgery, patients undergoing second eye surgery had lower levels of anxiety with higher levels of pain [28]. They postulated that the patients may be more attentive to the level of comfort during cataract surgery, rather than how successful the surgery would be. Perhaps, cataract surgery differs from intravitreal injections and retrobulbar blocks in that it is a longer procedure and the patients cannot visualize the instruments being directed towards them during the procedure because of the light of the microscope.

In recent years, there has been a trend towards PPV being performed in ambulatory surgery centers under retrobulbar block. In addition to no longer exposing the patient to the potential systemic complications of general anesthesia, this option also avoids unnecessary hospitalization, enables, in some countries, surgery to be performed without the presence of an anesthesiologist, and reduces surgical times, allowing for more patients to receive treatment [7]. This emphasizes the need to identify efficient ways of performing local anesthesia with as little pain as possible for the patient and to identify patients that may require extra attention, specifically prior to the retrobulbar block.

Ensuring patient cooperation during a retrobulbar block is of interest in order to maintain safety. As such, some surgeons routinely prescribe sedation such as propofol prior to retrobulbar blocks [29, 30]; however, propofol may carry its own potential lethal complications (although rare) [31]. As such, identifying patients that are most likely to benefit from anxiolytics or sedation prior to the retrobulbar block may be useful for the surgeon. In the current study, using the VASA score as a predictor of severe pain, an optimal cut-off point of VASA score >4 was identified. Therefore, it is reasonable to suggest that premedication may be considered in patients with moderate to severe anxiety prior to retrobulbar blocks and that a simple visual analog scale may be a sufficient tool to perform this task.

A limitation of this study is that though a clear correlation between anxiety and pain during retrobulbar blocks was demonstrated, it is unclear whether reducing the anxiety would indeed reduce pain. As such, future studies should evaluate whether anxiolytics and premedication benefit patients undergoing retrobulbar block, particularly those with moderate to severe anxiety.

## Figures and Tables

**Table 1:** Feelings experienced by patients during the procedure.

Parameter	Experienced (%)
Pain	22.9
Burning sensation	18.8
Pressure	52.1
Slight pinch	27.1
Pain compared to expectation	
Less	16.7
Equal	62.5
More	20.8

**Table 2:** Analysis of categorical preprocedural parameters and visual analogue scale (VAS) for pain scores.

Parameter	VAS scores (mean ± SD)	*p* value^*∗*^
Gender (M vs F)	3.4 ± 2.9 vs 3.7 ± 2.0	0.65
Native (Y vs N)	4.4 ± 2.9 vs 3.0 ± 2.2	0.10
Academic education (Y vs N)	3.5 ± 2.2 vs 3.5 ± 2.8	0.99
Retired (Y vs N)	3.4 ± 2.4 vs 3.6 ± 2.7	0.82
Past injections (Y vs N)	3.4 ± 2.2 vs 4.0 ± 3.7	0.64
Past operation (Y vs N)	3.5 ± 2.8 vs 3.5 ± 2.2	0.99
Preprocedural guidance (Y vs N)	3.2 ± 2.6 vs 4.1 ± 2.6	0.28
Anxiolytic before procedure (Y vs N)	3.6 ± 2.5 vs 3.3 ± 3.2	0.88

M: male; F: female; Y: yes; N: no; vs: versus. ^*∗*^Student's *t*-test applied.

**Table 3:** Multivariate analysis with visual analogue scale for pain (VAS) scores as the dependent variable and all variables that reached *p* < 0.30 in univariate analysis as independent variables.

Parameter	*R* ^2^ (%) (total = 21.2%)	*T* value	*p* value
VASA score (0–10)	18.9	2.68	0.01
Native (Y vs N)	1.8	−0.94	0.35
Preprocedural guidance (Y vs N)	0.56	−0.56	0.58

VASA: visual analog scale for anxiety; Y: yes; N: no; vs: versus.

**Table 4:** Binary logistic regression of different visual analog scale for anxiety (VASA) scores predicting severe visual analogue scale (VAS) for pain scores.

Anxiety level	Odds ratio	95% CI	*p*
Mild	0.10	0.01–0.94	0.02
Moderate	1.25	0.20–7.75	0.81
Severe	20.00	2.35–169.91	0.006

**Table 5:** Criterion values and coordinates of the receiver-operating characteristic curve for prediction of severe pain.

VASA criterion	Sensitivity	95% CI	Specificity	95% CI
≥0	100	54.1–100.0	0	0.0–8.4
>2	100	54.1–100.0	54.76	38.7–70.2
>3	83.33	35.9–99.6	66.67	50.5–80.4
>4^*∗*^	83.33	35.9–99.6	73.81	58.0–86.1
>5	66.67	22.3–95.7	90.48	77.4–97.3
>6	50	11.8–88.2	95.24	83.8–99.4
>7	33.33	4.3–77.7	100	91.6–100.0
>9	0	0.0–45.9	100	91.6–100.0

VASA: visual analogue scale for anxiety. ^*∗*^Youden's index assessed a cutoff of VASA score >4 for predicting severe pain.

## Data Availability

The data used to support the findings of this study are available from the corresponding author upon request.
